# Patient preference on once-daily oral versus injectable androgen deprivation therapy for Asian patients with advanced prostate cancer

**DOI:** 10.1007/s11255-024-04028-2

**Published:** 2024-03-21

**Authors:** Ho-Ming Chris Wong, Bernice Cheuk-Sin Cheung, Violet Wai-Fan Yuen, Jeremy Yuen-Chun Teoh, Peter Ka-Fung Chiu, Chi-Fai Ng

**Affiliations:** Division of Urology, Department of Surgery, Faculty of Medicine, SH Ho Urology Centre, The Chinese University of Hong Kong, Hong Kong SAR, China

**Keywords:** Patient preference study, Advanced prostate cancer, Androgen deprivation therapy, LHRH agonist, LHRH antagonist, Oral androgen deprivation therapy

## Abstract

**Purpose:**

The study aimed at investigating prostate cancer patients’ choice of androgen deprivation treatment (ADT) and possible factors that would affect their preferences of ADT.

**Methods:**

This was a single-centre cross-sectional study investigating the usage and preferences of ADT. Consecutives prostate cancer patients who were receiving injectable luteinizing hormone-releasing hormone (LHRH) agonist or antagonist were recruited from the prostate cancer clinic in a tertiary academic hospital. Patients who received bilateral orchidectomy or those who could not consent to the study were excluded. Disease characteristics, treatment information and patient background were documented. The survey collected information related to their change in ADT regimen, preferences on drug usage (routes and frequency of administration) and their reasons. A hypothetical set of three drug formularies was designed. Questions regarding patient preference and the contributing reasons raised in the format of questionnaire.

**Results:**

100 patients completed the survey. Most patients started with more frequent injections (3-monthly, 54%; 1-monthly, 38%) and switched to 6-monthly injections (89%) at the time of the survey. Primary reasons for the change were healthcare opinion (72%) and less frequent treatment (51%).

Three options of ADT (oral daily, 1-monthly and 6-monthly injection) with the same efficacies and side effect profile were offered: 61% preferred 6-monthly injection, 1% preferred 1-monthly injection and 38% preferred oral regimen. When patients were informed of lower cardiovascular side effects in 1-monthly injection or daily oral drug, patients’ preference was 56% (6-monthly), 6% (1-monthly), and 39% (oral). Patients with polypharmacy (more than 5 regular medications) were more inclined to choose injections (*p* = 0.025). Patient age, educational background, employment status, marriage status and disease status were not found to be statistically significant contributing factors to patient preference.

**Conclusion:**

6-monthly ADT injection was the preferred ADT despite greater cardiovascular risks. Among 1-monthly or daily oral LHRH antagonist, more patients prefer oral option. Convenience factor was highly valued.

## Introduction

Androgen deprivation treatment (ADT) has remained the backbone of the treatment for metastatic prostate cancer in the past decades [1]. Androgen deprivation can be achieved by three main methods: (1) bilateral surgical orchidectomy, (2) luteinizing hormone-releasing hormone (LHRH) agonist and (3) LHRH antagonist. Except in developing countries where many patients received surgical orchidectomy based on its cost-effectiveness, injectable medical ADT has long been the key component of the treatment of advance prostate cancer in developed regions like Hong Kong [2].

Aside from the readily available injectable medical ADT, oral LHRH antagonist has recently been used clinically with FDA approval [3]. Compared to the widely adopted injectables, the oral option presented itself as a medication of vast difference: different routes of administration, dosing schedule and systematic side effects. These are potential factors that might sway patient options when they are offered a choice. Understanding patient preferences had been shown to contribute significantly to medication adherence and hence better clinical outcomes [4]. There is a lack of literature that look into the factors that affect our patients’ choice of ADT formulation. In the following analysis, we would like to scrutinise the possible factors affecting patient’s choice of ADT.

## Methods and materials

### Overall design

This was a cross-sectional survey-based study performed in the Prince of Wales Hospital, a tertiary academic centre in Hong Kong SAR, China. Ethics approval had been granted by the local authority prior to the recruitment process. Consecutive prostate cancer patients who had at least received one dose of LHRH agonist or antagonist and who were able to consent were recruited. Those who received surgical bilateral orchidectomy and those who could not comprehend the study questions were excluded from the study. The target of recruitment was 100 patients. After obtaining informed consent, trained research staff assisted the patients to perform this cross-sectional survey. Information charted included patient’s demographics, disease status, treatment information, concomitant drug usage and their preference on the choice of ADT. Further details of the survey are described below.

### Survey

The written survey was started with documenting the patients’ demographics, co-morbidities and concomitant drug usage (number and route). The survey was written in Traditional Chinese. There were two parts to the survey. The first part was related to the current ADT use of the recruited subjects. They were asked to report on the type of injectable ADT they received at the time of survey and the type they were initially offered. Information was cross-checked by the research staff with the prescription history of ADT on the electronic system, after the completion of the questionnaire. If there were any discrepancy, reporting of ADT use was based on the actual formulary offered. It there was a change between the initial and the current ADT, subjects would be asked to choose one or more reasons that contributed to the change. The options of reason provided were:

- It feels like the new formulary is more efficacious.
- It feels like the new formulary comes with less side effects.
- The new formulary is more convenient.
- The new formulary needs less frequent injection.
- The new formulary feels more reversible compared to the old one.
- The new formulary is easier to remain compliant to.
- The new formulary brings less mental disturbance to me.
- The new formulary comes with better quality of life.
- There is fewer clinic follow-ups required when using the new formulary.
- Medical staff believed that the new formulary was a better choice for me.
- Family or friends believed that the new formulary was a better choice for me.

In the second part of the survey, the subjects were asked about their preference out of a set of three hypothetical ADT formularies.

Two tables listed the characteristics of the three involved hypothetical formularies. The first table listed out the characteristics of each formulary, namely, (1) the route of medication and (2) the frequency of clinic follow-up required (which could be in the form of a doctor consultation or injection clinic being run by urology nurses).

A. Medication A is an oral medication to be taken daily, requiring follow-up every few months.
B. Medical B is an injectable to be given every month as a subcutaneous injection, requiring monthly visit to the nursing clinic.
C. Medication C is an injectable to be given every 3 or 6 months, requiring follow-up every few months.

The second table discussed the potential adverse effects of the three captioned hypothetical formulae. The first column describes the cardiovascular complications in the format of major adverse cardiac and cerebrovascular event (MACCE) rates and the related death rates. The second column described the minor injection site morbidities of each formulary.

A. Medication A has around 50% fewer cardiovascular side effects than medication C, with no injection side effects as it is an oral medication.
B. Medication B has around 50% fewer cardiovascular side effects than medication C, with 40% of injection site side effects.
C. Medication C comes with comparatively more cardiovascular side effects, with 1% injection site side effects.

It was then followed by two general questions that read as follows. The patient was requested to choose one out of the three formularies for each question.

1. If the three formulae had the same treatment effects and side effects, which one would you choose as your preferred form of ADT?
2. If the three formulae had different side effects, which one would you choose as your preferred form of ADT?

Statistical analyses were performed with SPSS version 26 (IBM). Normally distributed continuous variables were expressed as means and standard deviations. Skewed variables were expressed as medians and interquartile ranges. Categorical variables were presented as counts and percentages. Two-sided *p* values of < 0.05 were considered statistically significant.

## Results

The mean age of the subjects was 72.0. At the time point when the survey was conducted, the median use of ADT duration was 24.1 ± 29.9 months. Out of the cohort, most of the patients in the state of metastatic hormone-sensitive prostate cancer (mHSPC) (66%), 18% were in castration-resistant prostate cancer (CRPC) status and the rest (16%) received ADT as part of the treatment for local disease (newly diagnosed or relapsing). The background characteristics of the patients included are reported in Table 1.

**Table 1:** Background characteristics of the cohort

	*N*	%
Median age ± SD	72	6.4
Working status		
Working	11	11
Retired/unemployed	89	89
Education		
Secondary education not completed	61	61
Completed secondary or vocational training	22	22
Completed tertiary education	15	15
Unknown	2	2
Partner status		
Married/partnered	92	92
Divorced/separated	4	4
Widowed	4	4
Living status		
Living with family/partner	92	92
Living alone	8	8
ECOG status		
0	83	83
1	16	16
2 or above	1	1
Requiring concomitant medication	91	91
Mean concomitant medication number ± SD	2.1	0.86
Requiring concomitant injection	4	4.4
Drug use frequency		
Daily	33	36.3
Two times per day	52	57.1
Three times per day	4	4.4
Four times or above per day	2	2.2
Needing assistance for drug use	5	5.5
Disease status at diagnosis		
Localised	70	70
Metastatic	30	30
Disease status at survey		
Localised under treatment	9	9
Localised disease relapse	7	7
mHSPC	66	66
CRPC	18	18

*mHSPC*  metastatic hormone-sensitive prostate cancer, *CRPC *castration-resistant prostate cancer

In the first part of the survey that looked into the ADT usage pattern, it was found that 38% of the patients started with 1-monthly ADT, 54% started with 3-monthly ADT and only 2% of patients began their treatment with 6-monthly ADT. At the time of the survey, a substantial number of patients had changed their ADT use already. 89% of the respondents were using a 6-monthly formulation. Contrastingly, the 1-monthly ADT was only adopted in 2% of the cohort. When asked about the reason behind the medication change, the most popular reason was “Medical staff believed that the new formulary was a better choice for me” (72%). The second common reason was “The new formulary needs less frequent injection” (51%). The details of the patient answer are listed in Table 2. The general opinion of the study population on choosing oral versus injectables was evaluated. When asked to choose between a potential oral and an injectable formula with a dosing frequency of at least 6 months, 59 patients would choose the oral formulary, and 41 would prefer the injectables. For those who preferred the oral choice, the most common reason chosen was “the medication appears to be more convenient”. On the other hand, for those who chose injectables, “the medication is easier to remain compliant to” was the most common reason (Table 3).

**Table 2:** The common reasons responsible for treatment changes from initial to current ADT

	*N*	%
Perception of treatment being more effective	2	2.2
Less side effects	3	3.4
Preferred route of administration	0	0.0
Requiring less frequent prescription	51	57.3
More reversibility	0	0.0
Allowing better compliance	0	0.0
Less psychological side effects	0	0.0
Better quality of life	0	0.0
Less frequent follow-up	17	19.1
More frequent follow-up	0	0.0
After receiving opinion from healthcare provider	72	80.9
After receiving opinion from family or friends	0	0.0

**Table 3:** General reasons between the choice of oral versus injectable medication of at least 6 months dosing interval

	*N*	%
Reason(s) if oral is chosen		
Perception of being more effectivef	1	1.7
Perception of disease being less severe	2	3.4
More convenient usage	46	78.0
More reversible effect	1	1.7
Not requiring needle	30	50.8
More flexible scheduling	22	37.3
Reason(s) if injectable is chosen		
Perception of being more effective	5	12.2
Perception of disease being less severe	0	0.0
More convenient usage	24	58.5
Less frequent treatment interval	25	61.0
Allowing better compliance	27	65.9
Having swallowing problem prohibiting oral medication	4	9.8

**Fig. 1:**
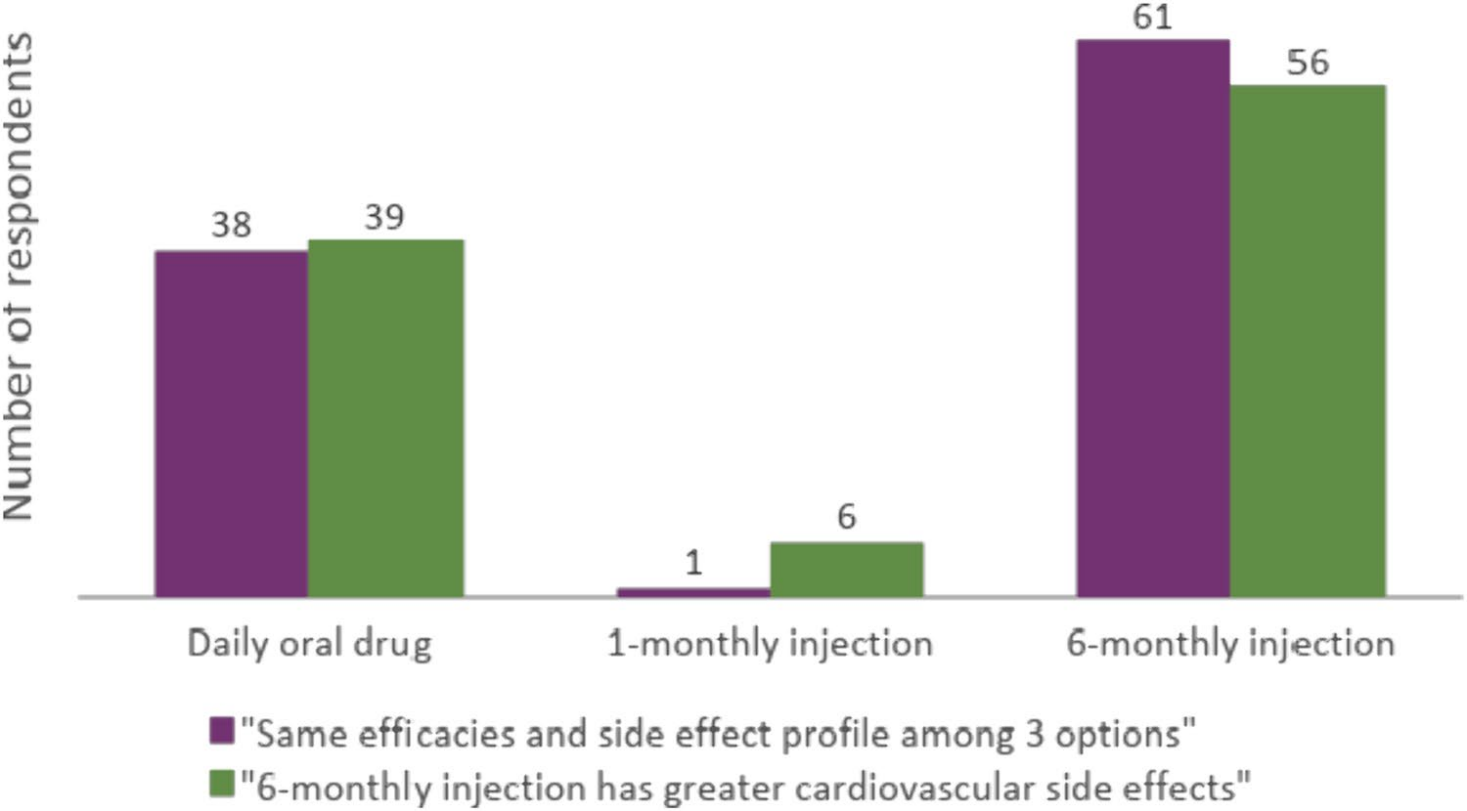
Patient’s preference of ADT based on the assumptions of similar or greater cardiovascular side effects resulting from 6-monthly injection

In the second part of the survey (hypothetical set of three formularies), when the patients were told that the side effects of the three formularies were considered equal, the majority of patients would opt for the 6-monthly injectable formulary (61%), 38% would choose the oral formulary and 1% would choose the 1-monthly injectable ADT. When asked to re-evaluate their choices if they knew that the 6-monthly injectable would result in higher cardiovascular side effects (compared to the 1-month injectable and the oral daily alternative), the results did not alter significantly. Most respondents would still opt for the 6-monthly injectable (56%) (Fig. 1). Exploratory analysis was conducted to determine what factors affect the patient’s preference. The patients who chose oral formulary were compared to those who chose injectable options. It was identified that those who had polypharmacy (defined as taking 5 or more oral medications per day) were more inclined to choose injectables (*p* = 0.025). Disease status (mHSPC or CRPC status compared to localised disease) was not shown to be a contributing factor for ADT preference. There were also no statistically significant differences observed in terms of education level, employment status or marital status (Table 4).

**Table 4:** Factors contributing to ADT preference

	*HR (CI 95%)*	*p value*
Having more than 5 concurrent medications	0.597	**0.025**
Working status (retired as reference)	0.667	0.741
Education (secondary education not completed as reference)	0.263	0.134
Partner status (married as reference)	0.471	0.97
Living status (living with family as reference)	0.793	0.891
ECOG status (ECOG status of 0 as reference)	0.492	0.758
Disease status at diagnosis (localised as reference)	0.442	0.756
Disease status at survey (localised under treatment as reference)	0.265	0.626

Bold value indicates statistical significance which is defined as *p* value < 0.05

*HR* hazard ratio, *CI *confidence interval

## Discussion

The current study examined patient preferences for injectable versus oral formulary for ADT in a group of advanced prostate cancer patients. The majority preferred a less frequent injectable. Less frequent dosing and healthcare opinion are the important reasons behind their choices. Currently, there was only limited conventional evidence that investigated patient preference for androgen deprivation. Nyman and colleagues looked into the factors that affected patient choice between surgical orchidectomy, bicalutamide and injectable ADT. They noted that the idea of being able to avoid injection or surgery and lower risk of impotence were the major factors driving their preferences. It was a more dated study, as orchidectomy and bicalutamide had already fallen out of favour for primary castration. It was also a study conducted with the Western population only [5]. De and colleagues published a study on patient concerns on ADT use, reporting that hot flushes and loss of libido were the worst perceived side effects. It was, however, a study conducted to examine whether intermediate-risk prostate cancer patients due for external beam radiotherapy would opt for additional ADT or not [6]. The clinical question and the patient subset were not comparable to our patient population. Evidence that aimed to evaluate the preferences of a group of advanced prostate cancer patients with ADT being universally indicated was lacking.

In our study, we found that a common reason behind any changes of ADT formulary was a less frequent injection. Quality of life (QoL) was postulated to be an important factor that swayed treatment decisions. Literature on breast cancer treatment preferences [7, 8] demonstrated that the perceived QoL played a significant role in formulary preference. The fact that a 6-monthly injection requires the least frequent clinic attendance and hence less impact to patients’ perceived QoL could be crucial in the decision process. Surprisingly, the reason of less side effects only accounted for 3% of the respondents that had a regimen change. Most of the patients switched from the 1-monthly LHRH antagonist to the 6-monthly LHRH agonist. Given injection site side effect was noted in up to 40% receiving 1-month injectable LHAH antagonist [9], it appeared that local injection side effect was less concerning than expected.

In the second part of the survey, even knowing that the hypothetical 6-monthly injectable coming would cause more cardiovascular side effects, little change was noted in the patient preferences with the majority still opting for the 6-monthly formulary. The variable differentiating these two questions was the perceived treatment-related cardiovascular risk. It is natural that that metabolic and cardiovascular implications of ADT are key concerns of physicians when it comes to offering ADT [10, 11]. Patient preference speaks otherwise in this regard. Despite knowing that the 6-monthly formulary came with more cardiovascular side effects, they were not swayed to opt for the 1-monthly injectable or the oral counterpart. This signifies they value a more convenient formulary over potential cardiovascular complications. Explanation is in twofold. First, the crude cardiovascular risk was too small for the recipients to perceive of. Second, frequent injection and its nuisance to daily life were more “pronounced”, whereas cardiovascular effect was comparatively more “silent”. Therefore, how physicians explain the pros and cons of the medications would be essential for patients to make an informed choice about ADT usage.

With the more recently available relugolix (oral LHRH antagonist) coming into play, advanced prostate cancer patients would be facing a more complex decision in ADT choices. In those with polypharmacy, adding another oral medication that requires daily dose would seem less attractive in this group of patients. In the second part of our survey, a substantial 39% of patients were more inclined for an oral prescription than injectable. This could mean that an oral formulary that totally evades the need of injection would still sound attractive for those with less co-morbidities.

The limitation of the current study should be highlighted. While our analysis focused primarily on patient preference regarding ADT use and that we included consecutive patients receiving ADT, the influence of combination treatment in mHSPC (including chemotherapy androgen receptor antagonist and even radiotherapy for oligometastatic mHSPC) was not elaborated. The disease stages of the current cohort were heterogeneous with the inclusion of localised disease alongside with metastatic patients. Further analysis of the effect of concomitant treatment within subgroups would most likely render our study underpowered to detect any statistically significant results. Further studies can be conducted to target on evaluating treatment preferences and the corresponding contributing factors in prostate cancer patients of different stages. Interaction between physician and patient preference was not evaluated. Often, physician preference may not correlate to patients’ choices in many circumstances because patient preferences were not easily understood fully [8]. Future investigations could potentially compare whether physician or nursing preferences are in line with patient preferences.

## Conclusion

Both 6-monthly injectable and daily oral formularies were popular choices among advanced prostate cancer patients. It was shown that convenience factors prevailed over medication side effects. The number of concomitant medication played a role in affecting patient decisions. The role of physician in facilitating patients to make an informed choice in face of different formulary options cannot be overstated.

## Data Availability

Sharing of data could be made available upon signing an agreement with The Chinese University of Hong Kong.
